# Membrane Disruption and Charge Neutralization Drive Synergy Between LL37-Derived Peptides and Polymyxins Against *E. coli*

**DOI:** 10.3390/biom16091324

**Published:** 2026-09-11

**Authors:** Wenxu Han, Nicholas James Watkins, Terri Anne Camesano

**Affiliations:** 1 Department of Chemical Engineering, Worcester Polytechnic Institute, 100 Institute Road, Worcester, MA 01609, USA; whan@wpi.edu; 2 Department of Materials Science and Engineering, Worcester Polytechnic Institute, 100 Institute Road, Worcester, MA 01609, USA; nicholas.watkins@saint-gobain.com; 3 Department of Chemical Engineering, University of Massachusetts Lowell, Lowell, MA 01854, USA

**Keywords:** antimicrobial peptide, cathelicidin LL37, LL37 fragments, combination strategy, zeta potential, outer membrane permeabilization, cytoplasmic membrane permeabilization

## Abstract

**Background/Objectives**: Antimicrobial peptides (AMPs) offer promising strategies for combating drug-resistant bacteria such as *Escherichia coli* (*E. coli*). This study evaluated the synergistic effects of the human-derived AMP LL37 and its fragments FK16 and FK13, in combination with polymyxin B, colistin, and vancomycin, against three *E. coli* strains in vitro. **Methods**: Because the outer membrane of *E. coli* acts as a barrier to antimicrobial agents, we assessed the membrane-permeabilizing activity of these AMPs. We further examined electrostatic interactions between the cationic AMPs and bacterial surfaces via zeta potential measurements. To simulate physiological conditions, we investigated how Mg^2+^ and Ca^2+^ ions, common in blood, stabilize the bacterial outer membrane and influence AMP activity. Additionally, we assessed cytoplasmic membrane permeabilization as a key mechanism of antibacterial action. Finally, to support clinical translation, we evaluated the cytotoxicity of the AMPs on human dermal fibroblasts. **Results**: Checkerboard assays revealed that the peptide–antibiotic combinations exerted synergistic effects against the tested *E. coli* strains in both MHB medium and cation-adjusted MHB medium, with FICI values ranging from 0.3125 to 0.5 and <0.375 to 0.5, respectively. All three AMPs (LL37, FK16, and FK13) exhibited strong outer membrane-permeabilizing activity. Zeta potential measurements revealed a correlation between membrane disruption and surface charge neutralization. The presence of Mg^2+^ and Ca^2+^ ions was found to stabilize the bacterial outer membrane. Cytoplasmic membrane permeabilization was confirmed as a key antibacterial mechanism. Cytotoxicity assays confirmed the safety profile of these AMPs on human cells. **Conclusions**: The study demonstrates that LL37 and its fragments enhance the activity of conventional antibiotics against *E. coli* through membrane disruption and charge neutralization, as evidenced by the FICI values (0.3125–0.5), membrane disruption and charge neutralization. While divalent cations in physiological conditions influence AMP efficacy, these peptides show potential for clinical application against *E.coli*.

## 1. Introduction

Antimicrobial resistance (AMR) has emerged as a major global public health crisis, driven in part by the misuse and overuse of antibiotics. AMR is currently responsible for approximately 700,000 deaths annually, a number projected to rise to 10 million by 2050 [1]. In 2017, the World Health Organization (WHO) released a list of 12 bacterial families urgently requiring new antibacterial strategies or antibiotics [2]. Among them, *Escherichia coli* (*E. coli*) is the most prevalent Gram-negative pathogen in both community and hospital settings, causing the highest absolute number of infections globally (including urinary tract infections, sepsis, and [3,4,5,6]), representing a primary healthcare problem [2,7]. Beyond its clinical significance, *E. coli* also serves as a well-characterized model organism for studying Gram-negative bacterial resistance and outer membrane permeability, offering a standardized and reproducible platform for initial efficacy testing of novel antimicrobial strategies.

As a Gram-negative bacterium, *E. coli* possesses an outer membrane that serves as a critical barrier, preventing many antibiotics from reaching intracellular targets. This membrane’s impermeability is reinforced by negatively charged lipopolysaccharide (LPS) molecules, which interact with divalent cations such as Mg^2+^ and Ca^2+^ to form stabilizing salt bridges, further reducing permeability [8]. Enhancing outer membrane permeability has been shown to improve antibiotic access and efficacy against Gram-negative bacteria [9].

Clinically, combination therapies are commonly employed to treat drug-resistant Gram-negative infections. For example, Zerbaxa (ceftolozane–tazobactam) and Zavicefta (ceftazidime–avibactam) are used to enhance antibacterial efficacy through synergistic mechanisms. However, these combinations may accelerate the emergence of resistance [10]. Notably, in 2023, the Centers for Disease Control and Prevention (CDC) reported a multi-state outbreak of Verona Integron-mediated Metallo-β-lactamase (VIM) Guiana-Extended Spectrum-β-Lactamase (GES)-producing carbapenem-resistant *Pseudomonas aeruginosa* (VIM-GES-CRPA), a strain resistant to both Zerbaxa and Zavicefta [11].

In this study, we explored the potential of combining conventional antibiotics with the human-derived antimicrobial peptide (AMP) LL37 and its structured fragments FK16 and FK13. LL37 is a 37-amino-acid cationic peptide that plays a key role in innate immunity [12], largely due to its amphipathic α-helical structure. However, this α-helicity is limited (approximately 70–80%) by a disordered N-terminal region [13], and LL37’s clinical use is further constrained by manufacturing costs and potential cytotoxicity [14]. To address these issues, truncated derivatives FK16 and FK13, which lack the disordered region, were developed. Compared to the full-length peptide, these shorter fragments retain potent antibacterial activity while offering several practical advantages, including reduced synthesis cost, broader-spectrum efficacy, and significantly lower cytotoxicity against mammalian cells [15,16,17]. The reduced toxicity is attributed to the removal of N-terminal hydrophobic residues, which have been associated with hemolytic and cytotoxic effects in the parent peptide [18].

We tested three antibiotics—vancomycin, colistin (polymyxin E), and polymyxin B—in combination with LL37, FK16, and FK13 against three clinical *E. coli* strains. Vancomycin inhibits cell wall synthesis by targeting lipid II but is generally ineffective against *E. coli* due to limited outer membrane penetration [19,20]. Vancomycin was selected as a model permeation probe to assess whether the combination strategy could facilitate the entry of otherwise impermeable antibiotics into *E. coli*. Previous work from our lab showed that LL37 enhances vancomycin efficacy against *P. aeruginosa* PAO1 by increasing membrane permeability [9,21]. We hypothesized that LL37 and its fragments could similarly sensitize *E. coli* to vancomycin.

Colistin and polymyxin B, both last-resort antibiotics for Gram-negative infections, act by disrupting the outer membrane [22]. However, resistance to these agents is rising, as seen in VIM-GES-CRPA strains [11], and their clinical use is further limited by dose-dependent nephrotoxicity. To preserve their efficacy and potentially reduce the required dosage, we evaluated whether combining polymyxins with AMPs could yield synergistic effects.

This study assessed the antibacterial activity of AMP-antibiotic combinations under both cation-adjusted and non-adjusted conditions, to examine how Mg^2+^ and Ca^2+^ modulate outer membrane stability. We also evaluated the role of exogenous LPS, AMP interactions with bacterial surfaces, and the relationship between charge neutralization and membrane permeabilization. Further, we analyzed cytoplasmic membrane disruption, vancomycin sensitization, and AMP-induced changes in permeability. Finally, to support clinical relevance, we assessed AMP cytotoxicity on human dermal fibroblasts.

## 2. Materials and Methods

### 2.1. Materials

**Figure 1:**
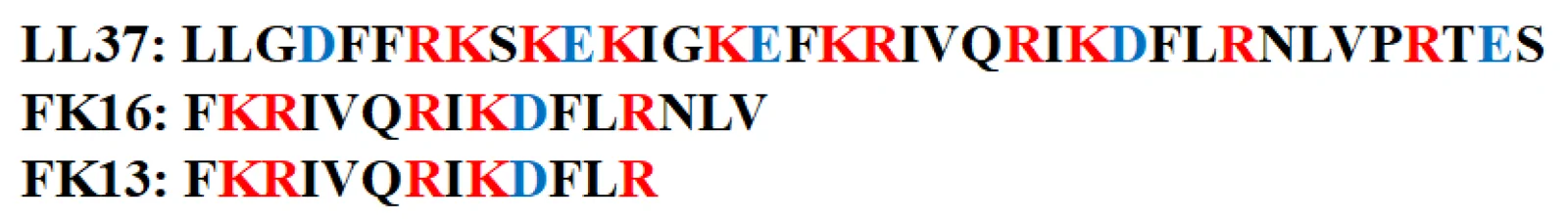
Sequence and charge distributions of LL37, FK16, and FK13. The red and blue letters represent the amino acid residues that carry positive and negative charges, respectively.

Three *E. coli* strains were studied in this research: Three clinically isolated uropathogenic *E. coli* (UPEC) strains (B37, B73, and B78), all derived from female patients with cystitis, were obtained from Dr. James Johnson (VA Medical Centers, Minneapolis, MN, USA) [23]. These well-characterized clinical isolates serve as representative models for evaluating antibacterial activity against pathogenic *E.coli* strains [24]. Bacteria were cultured in Mueller–Hinton broth (MHB) (Sigma-Aldrich, St. Louis, MO, USA) or cation-adjusted Mueller–Hinton broth (Sigma-Aldrich, St. Louis, MO, USA) and Mueller–Hinton agar (MHA) (Hardy Diagnostics CRITERION™, Santa Maria, CA, USA). Human dermal fibroblasts (ATCC CRL-2565) were cultured in Iscove’s Modified Dulbecco’s Medium (IMDM) (Thermo Fisher Scientific, Inc., Grand Island, NY, USA)) with supplement of 10% Fetal Bovine Serum (FBS) (Thermo Fisher Scientific, Inc., Grand Island, NY, USA) and 1% Penicillin-Streptomycin (10,000 U/mL, Thermo Fisher Scientific, Inc., Grand Island, NY, USA). Dulbecco’s phosphate-buffered saline (DPBS, 10×) was also purchased from Thermo Fisher Scientific, Inc. (Grand Island, NY, USA) RealTime-Glo™ MT Cell Viability Assay kit was purchased from Promega™ Corporation (Madison, WI, USA). LL37 (LLGDFFRKSKEKIGKEFKRIVQRIKDFLRNLVPRTES), FK16 (FKRIVQRIKDFLRNLV), and FK13 (FKRIVQRIKDFLR) (Figure 1) were purchased from Anaspec, Inc. (Fremont, CA, USA). Vancomycin, colistin, and polymyxin B, lipopolysaccharide (LPS) from *Escherichia coli* O55:B5, 4-(2-hydroxyethyl) piperazine-1-ethanesulfonic acid (HEPES) buffer solution (1 M in H_2_O), 1-N-phenylnaphthylamine (NPN), and propidium iodide (PI) were also procured from Sigma-Aldrich (St. Louis, MO, USA).

### 2.2. Broth Microdilution Assay

The minimum inhibitory concentrations (MICs) of LL37, FK16, FK13, and antibiotics were determined following Clinical and Laboratory Standards Institute (CLSI) guidelines [25]. AMPs and antibiotics were dissolved in sterile deionized water and stored at −80 °C until use.

For MIC testing, two-fold serial dilutions of antimicrobials were prepared in a 96-well polypropylene microtiter plate. *E. coli* colonies from Mueller–Hinton agar (MHA) plates were incubated overnight in MHB or cation-adjusted MHB (CMHB; 12.5 mg/L Mg^2+^, 25 mg/L Ca^2+^) at 37 °C in a Tissue Culture Roller Drum TC-7 (40 rpm) until log-phase growth. Bacterial suspensions were adjusted to a McFarland standard of 0.5 (~1.5 × 10^8^ CFU/mL), then diluted to a final concentration of 5 × 10^5^ CFU/mL. A 90 μL aliquot of the suspension was added to each well of a 96-well plate, followed by incubation at 37 °C for 20 h without shaking. MICs were recorded as the lowest antimicrobial concentrations inhibiting visible bacterial growth.

To assess the protective effects of LPS, LPS was dissolved in DI water to 1 mg/mL. In a 96-well plate, 10 μL of two-fold serial antimicrobial dilutions and 10 μL of LPS (1 mg/mL) were combined, followed by the addition of 80 μL of *E. coli* suspensions to reach a final concentration of 5 × 10^5^ CFU/mL. MICs in the presence of LPS were determined as described above. All experiments were performed with three independent biological replicates, each comprising three technical replicates.

### 2.3. Checkerboard Assay

The checkerboard assay was used to determine synergistic AMP-antibiotic combinations [9]. To reach a 1:1 mix of AMP and antibiotic, 10 μL of different concentrations of AMPs and antibiotics were prepared in a 96-well plate. 80 μL of *E. coli* suspensions were added to a 96-well plate to reach a final concentration of 5 × 10^5^ CFU/mL. The 96-well plate was incubated for 20 h at 37 °C without shaking. The synergistic effects can be determined by calculating the fractional inhibitory concentration index (FICI) [9]. The formula for calculating FICI is:

$$\frac{\left(MIC\;of\;drug\;A\;in\;combination\right)}{\left(MIC\;of\;drug\;A\;alone\right)}+\frac{\left(MIC\;of\;drug\;B\;in\;combination\right)}{\left(MIC\;of\;drug\;B\;alone\right)}$$

Synergy can be defined when FICI ≤ 0.5 [9]. The experiments were repeated three times independently.

### 2.4. Neutralization Process

Bacteria–antimicrobial interactions were assessed by measuring zeta potential changes in three *E. coli* strains upon antimicrobial addition. The net charges of LL37, FK16, FK13, polymyxin B, vancomycin, and ampicillin are shown in Table 1.

*E. coli* cultures were grown to log phase at 37 °C, centrifuged at 4000 rpm for 5 min, washed three times with DI water, and diluted to 1 × 10^6^ CFU/mL. Suspensions were loaded into disposable folded capillary cells, and zeta potential was measured using a Zetasizer Nano-ZS ZEN 3600 (Malvern Instruments, Inc., Malvern, UK)). Changes in zeta potential with increasing antimicrobial concentrations were recorded. Measurements were performed in triplicate.

To evaluate electrostatic interactions between lipopolysaccharide (LPS) and antimicrobial peptides (AMPs), changes in zeta potential were measured following AMP treatment. LPS from *E. coli* O55:B5 was dissolved in deionized (DI) water to a final concentration of 100 μg/mL and used immediately without sonication. Zeta potential measurements were performed using the same protocol described previously, and shifts in surface charge were recorded at increasing concentrations of LL37, FK16, and FK13. All measurements were conducted in triplicate.

To assess the role of divalent cations (Mg^2+^ and Ca^2+^) in modulating electrostatic interactions with *E. coli* and LPS, bacterial cultures were prepared by growing the three *E. coli* strains in CMHB overnight at 37 °C until logarithmic phase. Cells were then harvested by centrifugation and resuspended in cation-adjusted DI water (final concentrations: 12.5 mg/L Mg^2+^ and 25 mg/L Ca^2+^) to 1 × 10^6^ CFU/mL. LPS was also dissolved in the same cation-adjusted DI water at 100 μg/mL. Zeta potential measurements were performed for each strain and for LPS in the presence of divalent cations. Each condition was measured independently in triplicate.

### 2.5. Outer Membrane Permeability Test

Outer membrane permeability changes in three *E. coli* strains were assessed by measuring the uptake of the fluorescent probe NPN, using a previously published method [27]. NPN was dissolved in acetone to reach a final concentration of 2 mg/mL.

*E. coli* strains were cultured in MHB at 37 °C to logarithmic phase and diluted to an optical density of OD_600_ = 0.2. Two milliliters of each bacterial suspension were incubated with LL37, FK16, FK13, polymyxin B, vancomycin, or ampicillin at a final concentration of 25.6 μg/mL for 1 h at 37 °C. Following treatment, cells were centrifuged at 8000 rpm for 5 min and washed twice with GHEPES buffer (pH 7.25; 5 mM HEPES, 5 mM glucose).

NPN (40 μL of the 2 mg/mL stock solution) was added to the washed *E. coli* suspensions, which were then incubated in the dark at room temperature for 30 min. Fluorescence was measured using an F-4500 Fluorescence Spectrophotometer (Hitachi, Hitachi High-Technologies Co., Tokyo, Japan) under the following settings: excitation at 350 nm, emission at 420 nm, and a PMT voltage of 700 V.

The increased outer membrane permeability can be calculated as the percentage of NPN uptake:

NPN uptake (%) = (F_Antimicrobial_ − F_C_)/(F_C_) × 100%

F_Antimicrobial_ is the fluorescence of *E. coli* strains with the addition of antimicrobials; F_C_ is the fluorescence of *E. coli* strains with the addition of DI water and serves as the control group. The experiments were repeated three times independently.

To evaluate the protective and stabilizing effects of Mg^2+^ and Ca^2+^ on the *E. coli* outer membrane, bacterial strains were cultured overnight in CMHB at 37 °C and diluted to an OD_600_ of 0.2. Antimicrobials were added to the suspensions at the same final concentration as described above (25.6 μg/mL) and incubated for 1 h at 37 °C. Following treatment, the bacterial suspensions were centrifuged and washed with GHEPES buffer as previously described. NPN fluorescence was then measured to assess outer membrane permeability in the presence of divalent cations.

### 2.6. Sensitization Assay

The ability of AMPs to sensitize *E. coli* to vancomycin was evaluated by measuring changes in vancomycin MIC following AMP pretreatment. *E. coli* strains were cultured in MHB at 37 °C to logarithmic phase, then diluted to 1 × 10^6^ CFU/mL. Bacteria were incubated with LL37, FK16, or FK13 at half their respective MICs for 1 h.

Following pretreatment, two-fold serial dilutions of vancomycin were prepared in a 96-well polypropylene plate. Control groups received no AMP pretreatment. Treated bacterial suspensions were diluted and 90 μL was added to each well, yielding a final inoculum of 5 × 10^5^ CFU/mL.

Plates were incubated at 37 °C for 20 h without shaking. Vancomycin MICs were then determined by visual inspection as the lowest concentration with no visible bacterial growth.

### 2.7. Cytoplasmic Membrane Permeability Test

To investigate the cytoplasmic membrane-disrupting properties of LL37 and its fragments, membrane permeabilization was assessed using propidium iodide (PI), following a previously published protocol [27]. PI was dissolved in DI water at a concentration of 1 mg/mL.

*E. coli* strains were cultured in MHB at 37 °C to logarithmic phase, diluted to OD_600_ = 0.2, and treated with LL37, FK16, FK13, or vancomycin (25.6 μg/mL) for 1 h at 37 °C. A negative control group was incubated in DI water without antimicrobials.

After treatment, cells were centrifuged at 8000 rpm for 5 min and washed twice with GHEPES buffer (pH 7.25). The bacterial pellets were then resuspended and stained with 20 μL of PI solution (1 mg/mL), followed by incubation in the dark at room temperature for 30 min.

Fluorescence was measured using an F-4500 Fluorescence Spectrophotometer (Hitachi, Hitachi High-Technologies Co., Tokyo, Japan) with the following settings: excitation wavelength 535 nm, emission wavelength 620 nm, and PMT voltage 700 V. All measurements were performed in triplicate.

### 2.8. Cytotoxicity Assay

Cytotoxicity of LL37, FK16, and FK13 was evaluated using human dermal fibroblasts (HDFs). Cells were cultured in flasks at 37 °C in a humidified incubator with 5% CO_2_ until approximately 70% confluence, as confirmed by optical microscopy.

The cytotoxicity of the peptides was evaluated across a concentration range of 0.25–256 μg/mL. AMP solutions were prepared at various concentrations, and 5 μL of each was added to wells of a 96-well tissue culture plate. HDFs were seeded into the plate at a density of 3000 cells/well in 45 μL of Iscove’s Modified Dulbecco’s Medium (IMDM). Cells treated with deionized (DI) water served as the negative control.

Plates were incubated for 24 h at 37 °C with 5% CO_2_. Following incubation, 50 μL of 2× RealTime-Glo™ reagent was added to each well, and the plate was incubated for an additional 30 min. Luminescence was measured using a PerkinElmer Victor^3^ multilabel plate reader. Cell viability was quantified by comparing luminescence signals from AMP-treated wells to those from the control group.

## 3. Results

### 3.1. Anti-E. coli Effect of AMPs and Antibiotics

As expected, polymyxin B and colistin showed lower MICs when compared to other antimicrobials for the three *E. coli* strains in this study (Table 2). Vancomycin targets lipid II, a precursor peptide located within the cell, which is vital for cell wall synthesis. However, the outer membrane of Gram-negative bacteria acts as a barrier that hinders access of vancomycin to this target [9]. Vancomycin exhibited the highest MICs to three *E. coli* strains, indicating poor antibacterial effects (Table 2).

LL37, FK16, and FK13 exhibited antibacterial effects against the three *E. coli* strains, with FK16 demonstrating the best antibacterial efficacy among the three AMPs (Table 2). The structural differences among the three AMPs may contribute to their different antibacterial effects. FK16 is more structured than LL37, which may account for the increased efficacy, as α-helical secondary structure is crucial for AMPs to show antibacterial effects [13]. FK16 also contains N30, L31, and V32 residues, which play important roles in determining the antibacterial efficacy of LL37, thus resulting in better antibacterial effects for FK16 compared to FK13 [15]. Our results correspond to previous research indicating that the antibacterial efficacy of FK13 is decreased due to the absence of N30, L31, and V32 residues [15].

The antibacterial efficacy of these antimicrobials was also assessed in CMHB medium containing Mg^2+^ and Ca^2+^ ions (Table 3). The concentrations of Mg^2+^ and Ca^2+^ ions mimic those found in human blood. Mg^2+^ and Ca^2+^ ions can protect and stabilize the outer membrane through the creation of salt bridges with negatively charged LPS. Cationic antimicrobials need to compete with and displace Mg^2+^ and Ca^2+^ ions that are bound to LPS in order to penetrate the outer membrane and achieve antibacterial efficacy [28].

The MICs of all antimicrobials increased against the three *E. coli* strains, except for colistin, which remained unchanged against strain B73, indicating the protective effects of Mg^2+^ and Ca^2+^. The MICs for polymyxin B and colistin generally increased, suggesting outer membrane stabilization by these cations; however, polymyxins maintained low MICs, demonstrating their ability to penetrate the membrane effectively. Vancomycin MICs doubled across all strains, indicating that Mg^2+^ and Ca^2+^ may reduce outer membrane permeability.

The presence of Mg^2+^ and Ca^2+^ ions showed a significant influence on the antibacterial effects of AMPs. A 4-fold to 16-fold increase in MIC was observed in the presence of the cations. The low salt tolerance of LL37 and its analogs may constrain their clinical potential [13]. FK16 displayed the lowest MICs among the three AMPs (except for B37, where FK16 exhibited the same MIC as FK13), thereby indicating the better antibacterial potential of FK16.

LPS is an important component of the outer membrane and influences the outer membrane permeability, thereby hindering the penetration of antimicrobials [29]. To investigate the impact of LPS on the antibacterial effects of AMPs, we assessed the MICs of AMPs against three *E. coli* strains in the presence of exogenous LPS (100 μg/mL) (Table 4). The concentration of 100 μg/mL exogenous LPS was chosen as a saturation condition to evaluate the maximal inhibitory effect of LPS on AMP activity, rather than to reflect physiological LPS levels at infection sites. The MICs of all three AMPs increased several-fold. The exogenous LPS significantly hindered the antibacterial effects of LL37 and FK13, resulting in increased MICs of >256 μg/mL and 128 μg/mL, respectively. The MICs of FK16 also exhibited 4-fold to 8-fold increases; however, FK16 maintained the lowest MICs compared to the other two AMPs. The increased MICs of AMPs in the presence of exogenous LPS suggest that AMPs not only bind to the LPS at membrane surfaces but also bind to exogenous LPS in solution.

### 3.2. AMP-Antibiotic Combination Effects

LL37, FK16, and FK13 were combined with vancomycin, polymyxin B, and colistin to evaluate potential synergistic effects against three *E. coli* strains in MHB medium (Table 5). Synergy was more frequently observed when AMPs were paired with membrane-targeting antibiotics (polymyxin B and colistin) than with vancomycin, which targets lipid II. While AMPs are often combined with intracellular-targeting antibiotics to enhance uptake, our findings suggest that pairing two membrane-active agents may also yield promising synergies, warranting further investigation of such combinations. The MIC values of each antimicrobial alone and in combination, along with the corresponding FICI values, are provided in Table S1.

AMP-antibiotic combinations were also tested in CMHB medium to assess the influence of Mg^2+^ and Ca^2+^ ions (Table 6). Despite the protective effects of these cations, AMP-polymyxin B combinations continued to show synergy against all three *E. coli* strains. AMP-colistin combinations were synergistic against B78 and B37 but not B73. In contrast, AMP-vancomycin combinations exhibited synergy only in two cases against B37 and showed no synergy against the other strains. These results suggest that, even in the presence of divalent cations that stabilize the outer membrane, combinations with polymyxin B and colistin retain greater synergistic potential than those with vancomycin. The MIC values of each antimicrobial alone and in combination, along with the corresponding FICI values, are provided in Table S2.

### 3.3. Neutralization Process

**Figure 2:**
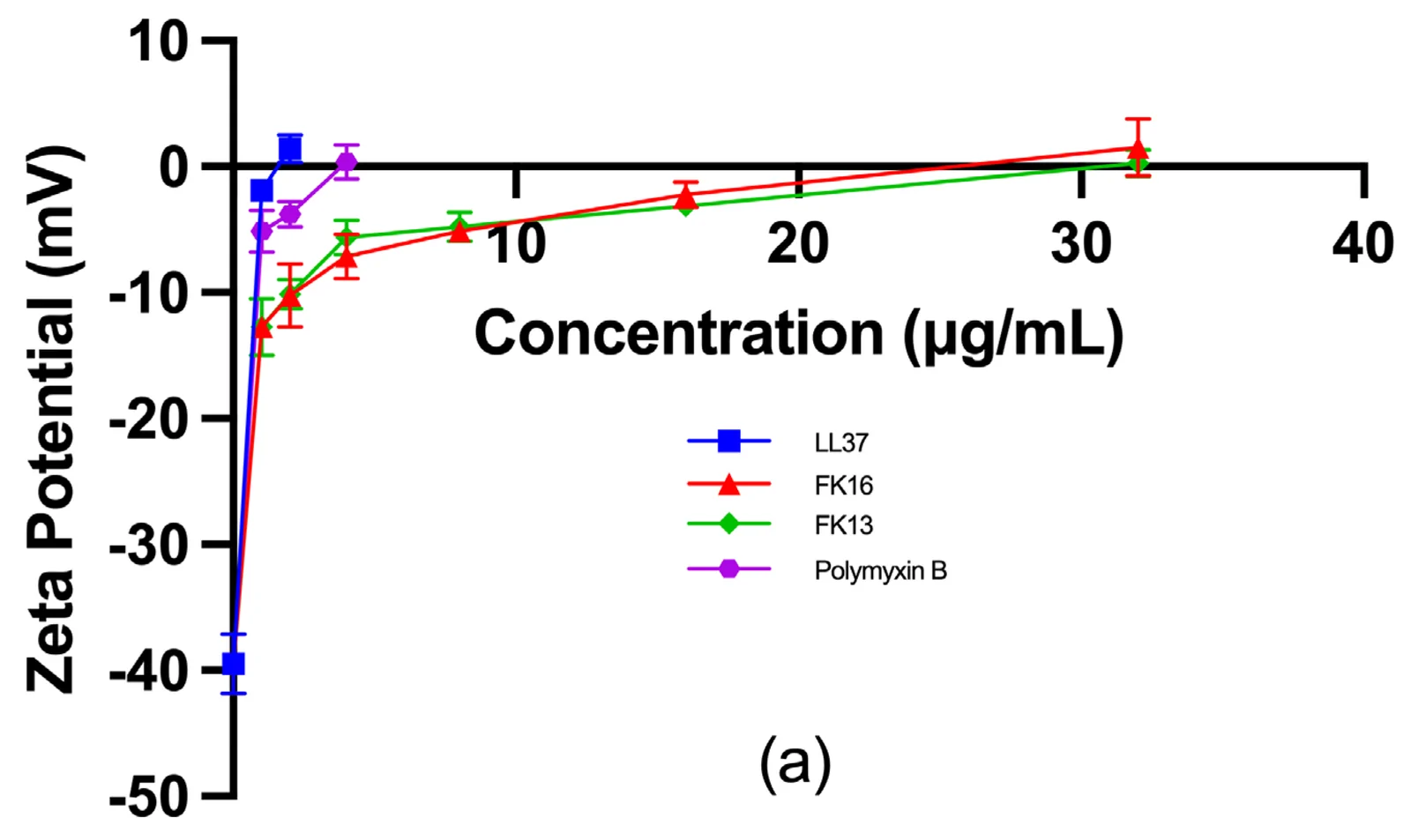

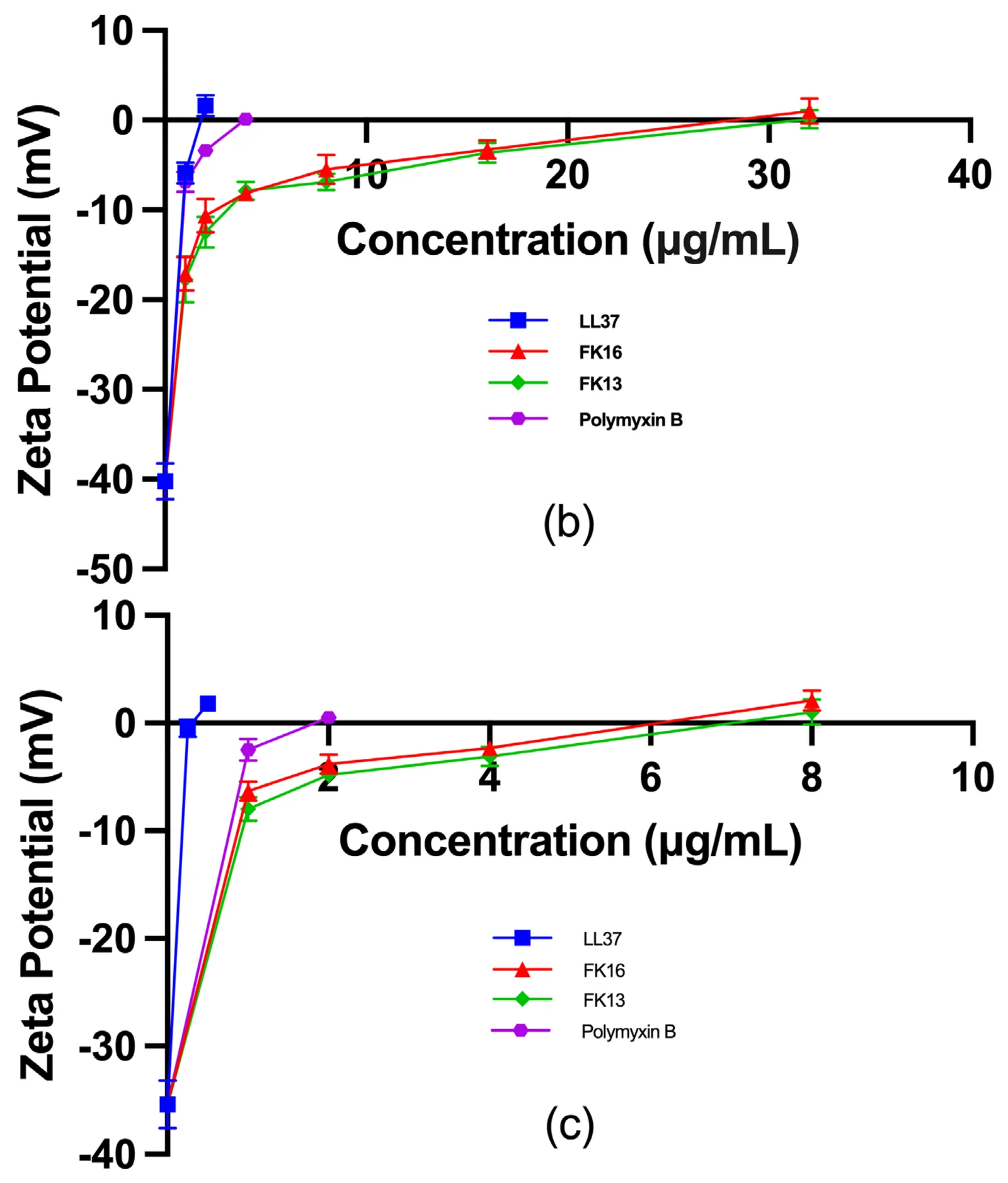
Changes in zeta potential of (**a**) *E. coli* B78, (**b**) *E. coli* B37, (**c**) *E. coli* B73 with addition of LL37, FK16, FK13, and polymyxin B. The error bars indicate the standard deviations after three independent experiments.

The zeta potentials of *E. coli* strains B78, B37, and B73 were −39.5 mV, −40.2 mV, and −35.4 mV, respectively (Figure 2). As expected, the concentration of each AMP required to neutralize surface charge correlated with its net positive charge (Table 1). LL37, with the highest net charge (+6), required the lowest concentration for neutralization. Polymyxin B (+5) required slightly higher concentrations, while FK16 and FK13 (both +4) showed similar neutralization profiles across all strains.

**Figure 3:**
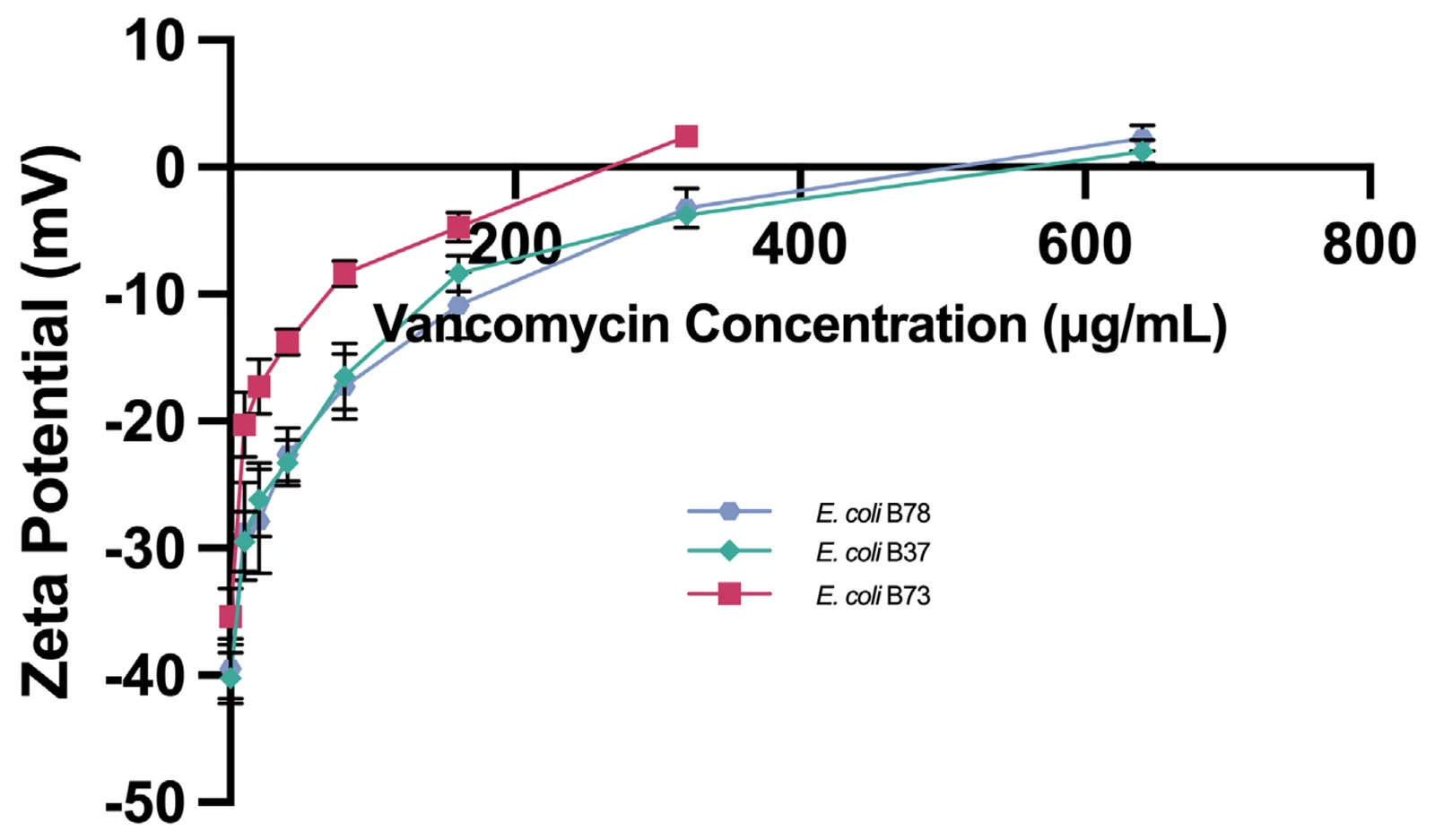
Changes in zeta potential of *E. coli* B78, *E. coli* B37, *E. coli* B73 with addition of vancomycin. The error bars indicate the standard deviations after three independent experiments. The net charge for vancomycin is +0.83 mV [26].

**Figure 4:**
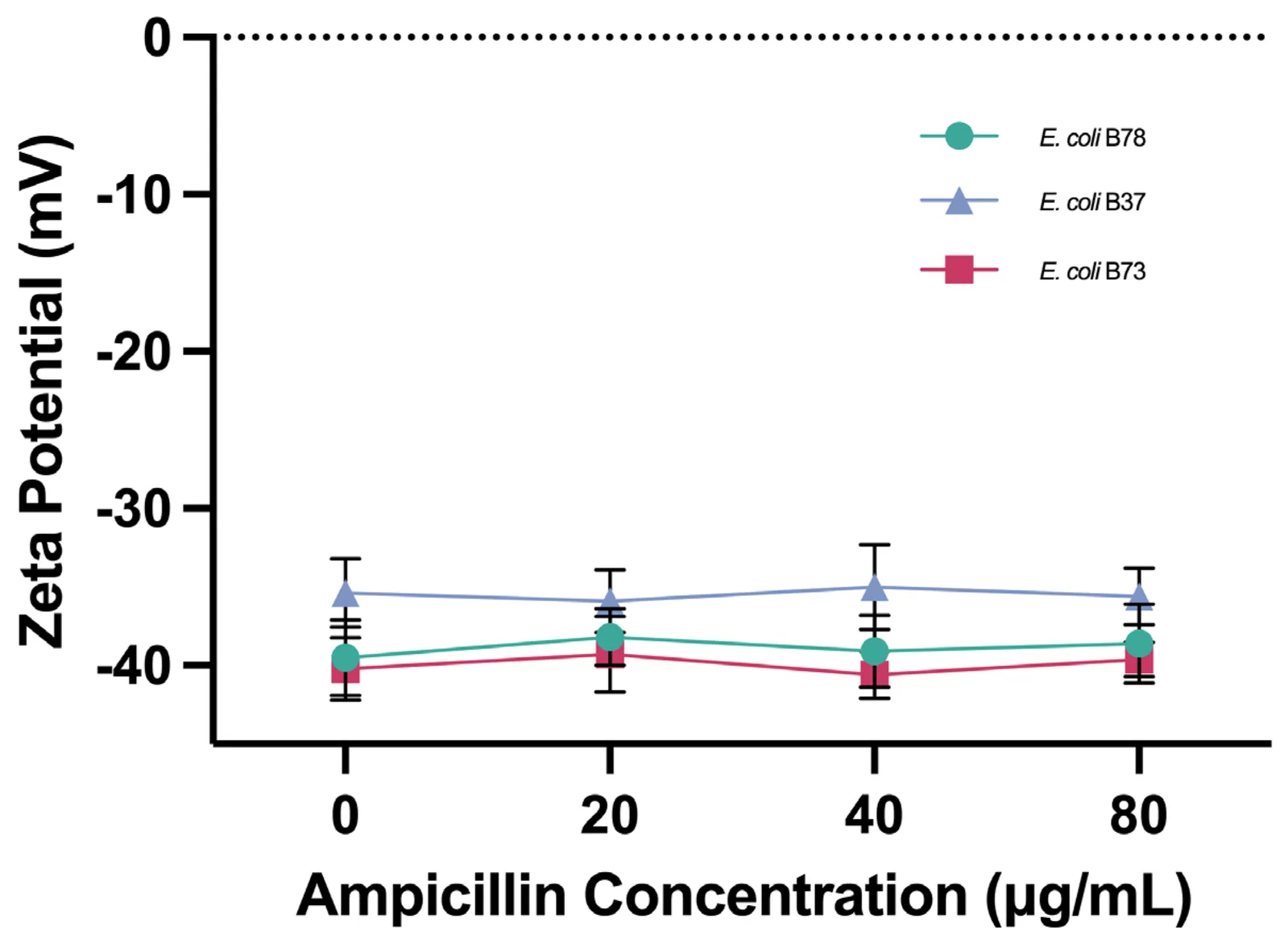
Changes in zeta potential of *E. coli* B78, *E. coli* B37, and *E. coli* B73 with addition of ampicillin. The error bars indicate the standard deviations after three independent experiments. The net charge for ampicillin is 0 mV.

To further examine the relationship between antimicrobial charge and neutralization capacity, we measured zeta potential shifts following the addition of vancomycin and ampicillin (Figure 3 and Figure 4). Vancomycin, with a lower net charge (+0.83), required higher concentrations to neutralize *E. coli* compared to cationic AMPs and polymyxin B. B73 required less vancomycin for neutralization than B37 and B78, likely due to its less negative baseline zeta potential. In contrast, ampicillin (net charge = 0) failed to neutralize the negative surface charges of any strain. Vancomycin, despite its lack of antibacterial activity against *E. coli*, exhibited a greater capacity to neutralize the bacterial surface charge compared to ampicillin, which is likely attributable to its positive net charge (+0.83 mV) versus the neutral charge of ampicillin. This electrostatic effect does not correlate with antibacterial efficacy. These findings reinforce that charge neutralization is governed primarily by the net positive charge of the antimicrobial and the initial surface charge of the bacterial strain.

**Figure 5:**
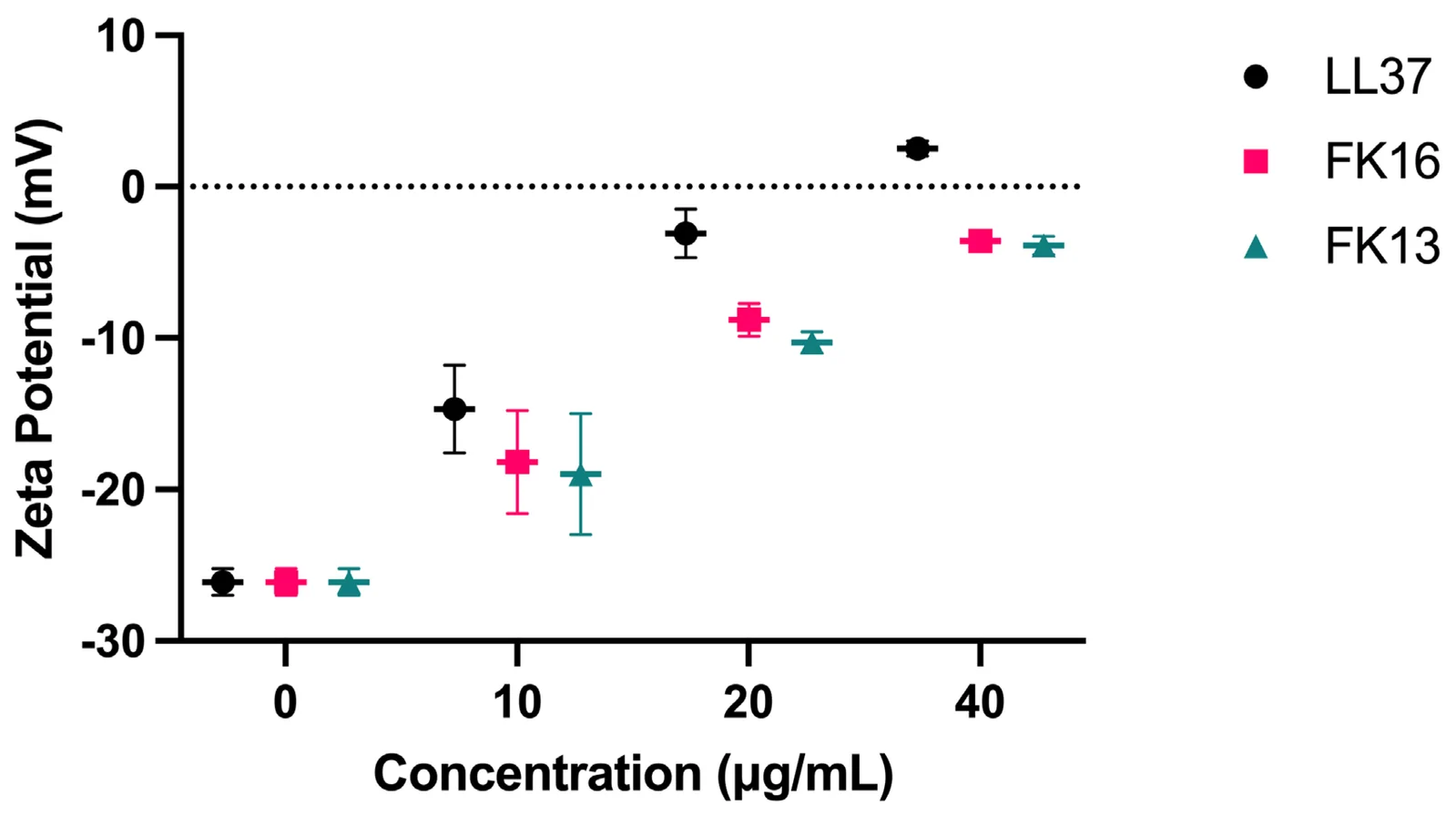
Changes in zeta potential of LPS (100 μg/mL) with addition of AMPs. The error bars indicate the standard deviations after three independent experiments.

The presence of exogenous LPS reduced the antibacterial activity of all three AMPs (Table 4). We hypothesized that this was due to electrostatic binding of the cationic peptides not only to the bacterial surface but also to free LPS in solution, effectively sequestering the AMPs. To test this, we repeated the zeta potential measurements using LPS solutions (100 μg/mL) with increasing concentrations of LL37, FK16, and FK13 (Figure 5). All three peptides increased the zeta potential of the LPS solutions, confirming their ability to bind LPS. LL37 showed the strongest neutralizing effect, while FK16 and FK13 performed similarly, consistent with their matched net charges.

Although the initial zeta potential of the LPS solutions was less negative than that of whole bacteria, higher AMP concentrations were required for neutralization. Moreover, the differences in required concentrations between LL37 and its fragments were smaller in LPS solutions than in bacterial suspensions. These observations suggest that AMPs interact differently with free LPS than with LPS embedded in the outer membrane. One possible explanation is that AMP-induced aggregation of free LPS may limit access to all negatively charged binding sites, leading to only partial zeta potential changes [30]. Nevertheless, AMPs such as LL37 are known to dissociate LPS aggregates and neutralize endotoxins, highlighting their potential role in anti-sepsis applications [31,32].

### 3.4. Outer Membrane Permeabilizing Effects

**Figure 6:**
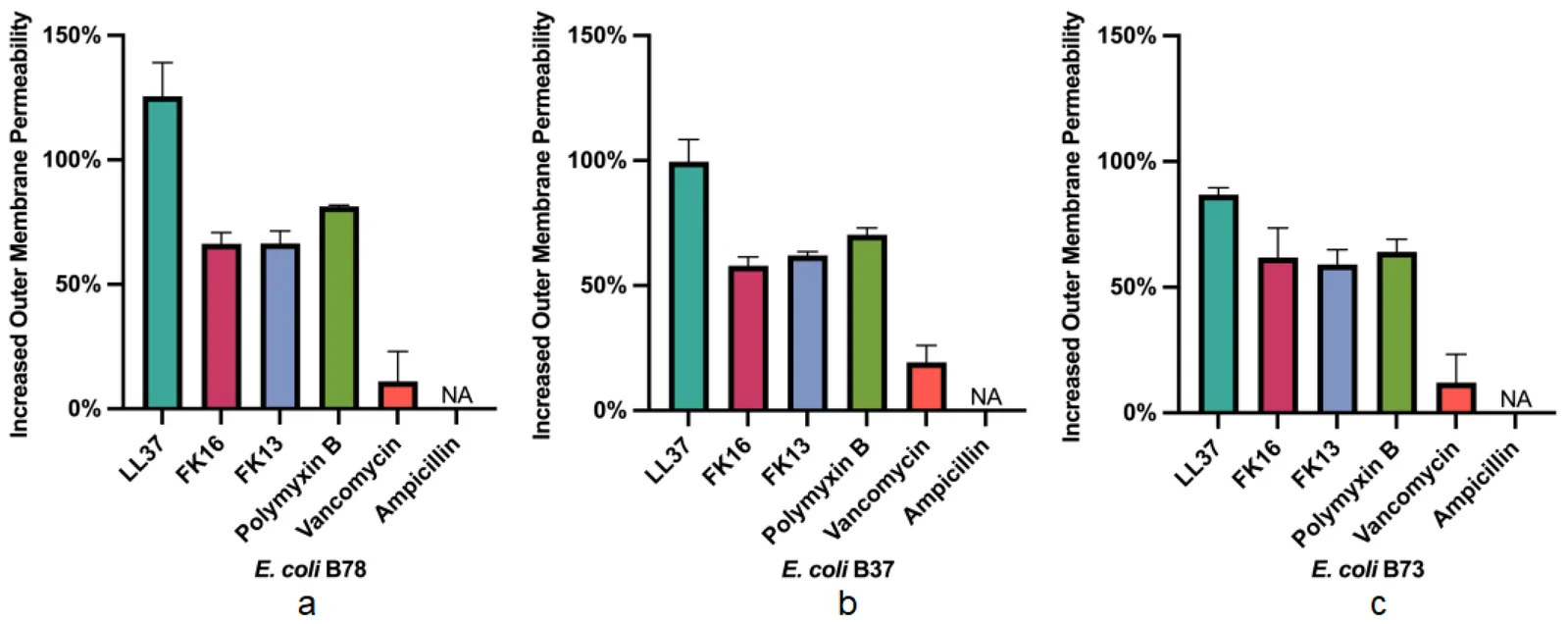
Outer membrane permeabilizing properties of antimicrobials against (**a**) *E. coli* B78, (**b**) *E. coli* B37, (**c**) *E. coli* B73 in the absence of Mg^2+^ and Ca^2+^ ions. The error bars indicate the standard deviations after three independent experiments.

Outer membrane permeabilization was assessed via uptake of the hydrophobic fluorescent probe NPN, which increases in fluorescence upon entering bacteria. Each cationic antimicrobial was shown to increase the outer membrane permeability of the three *E. coli* strains (Figure 6).

LL37 showed the strongest outer membrane permeabilizing activity, followed by polymyxin B, with FK16 and FK13 displaying similar but somewhat weaker effects. Vancomycin exhibited minimal permeabilization, and ampicillin had no detectable effect. These results suggest that outer membrane permeabilization generally correlates with the net positive charge of the antimicrobial agent.

**Figure 7:**
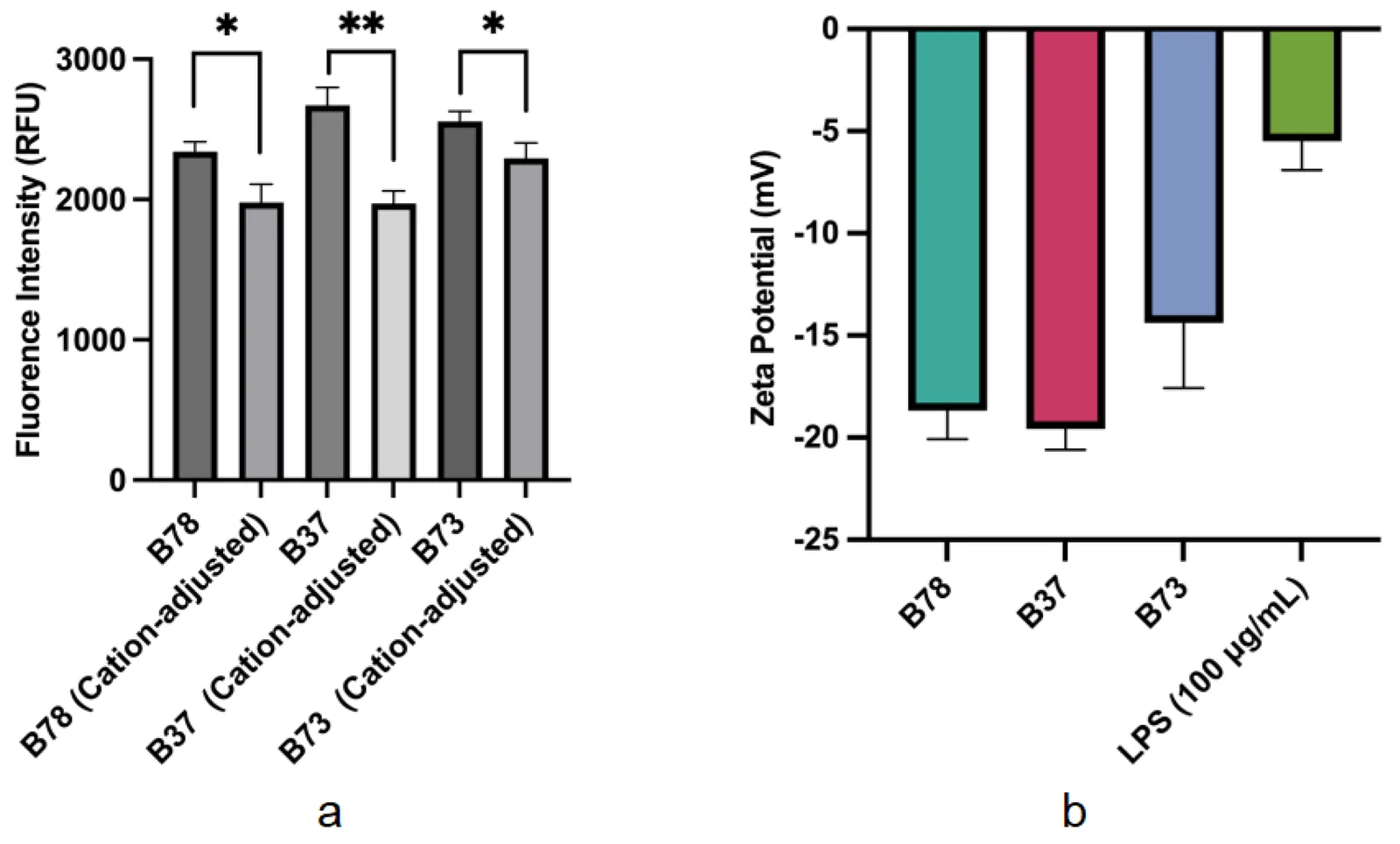
(**a**) Outer membrane permeability of three *E. coli* strains in the presence or absence of Mg^2+^ and Ca^2+^ ions, * stands for *p* ≤ 0.05, ** stands for *p* ≤ 0.01, (**b**) binding performance of three *E. coli* strains and LPS (100 μg/mL) in the presence of Mg^2+^ and Ca^2+^ ions. The error bars refer to the standard deviations after three independent experiments.

To assess the stabilizing effects of Mg^2+^ and Ca^2+^, we compared outer membrane permeability in the presence and absence of these ions. At concentrations of 12.5 mg/L Mg^2+^ and 25 mg/L Ca^2+^, all three *E. coli* strains showed reduced membrane permeability (Figure 7a). Zeta potential measurements also increased for both the bacterial cells and LPS solutions (100 μg/mL) in the presence of these cations (Figure 7b), consistent with the formation of stabilizing salt bridges between divalent cations and LPS.

**Figure 8:**
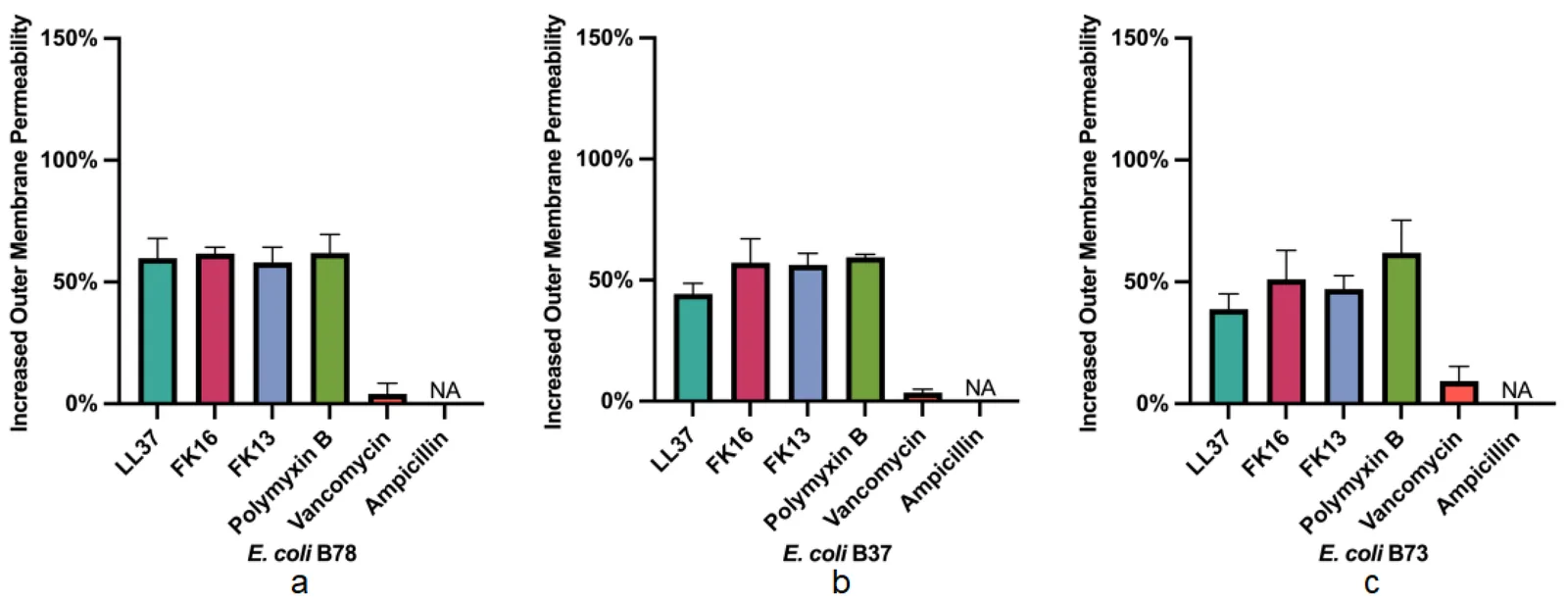
Outer membrane permeabilizing properties of antimicrobials against (**a**) *E. coli* B78, (**b**) *E. coli* B37, (**c**) *E. coli* B73 in the presence of Mg^2+^ and Ca^2+^ ions. The error bars refer to the standard deviations after three independent experiments.

In the presence of Mg^2+^ and Ca^2+^, the outer membrane permeabilizing activity of cationic antimicrobials was reduced (Figure 8), likely contributing to the higher MICs observed in CMHB. LL37’s activity declined most noticeably, while polymyxin B remained the most effective. FK16 and FK13 showed similar, moderate activity, and vancomycin remained weakly permeabilizing. As expected, ampicillin had no effect.

### 3.5. Sensitization of E. coli Strains to Vancomycin

**Figure 9:**
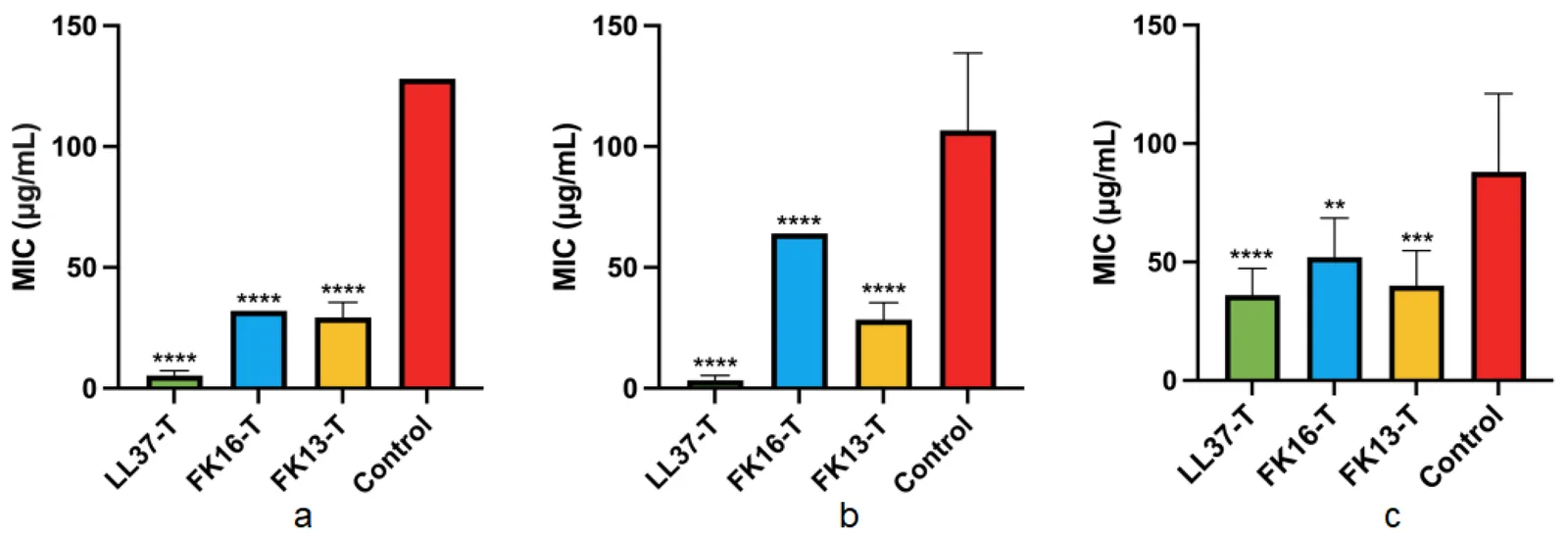
MICs of vancomycin against *E. coli* strains after peptide treatment. (**a**) *E. coli* B78, (**b**) *E. coli* B37, (**c**) *E. coli* B73. The control groups were operated under the same conditions except for the treatment with peptides. The error bars indicate the standard deviations after three independent experiments; data without error bars indicate that the SD is too small to be seen. ** stands for *p* ≤ 0.01, *** stands for *p* ≤ 0.001, **** stands for *p* ≤ 0.0001.

All three AMPs lowered the MICs of vancomycin against the three *E. coli* strains (Figure 9). LL37 pretreatment decreased vancomycin MICs by 24- to 32-fold in B78 and B37 (Table 7), consistent with its strong outer membrane permeabilization. The results suggest that increased outer membrane permeability can suppress the intrinsic resistance of *E. coli* strains to vancomycin by enhancing the ability for vancomycin to reach its lipid II target.

### 3.6. Cytoplasmic Membrane-Permeabilizing Effects

**Figure 10:**
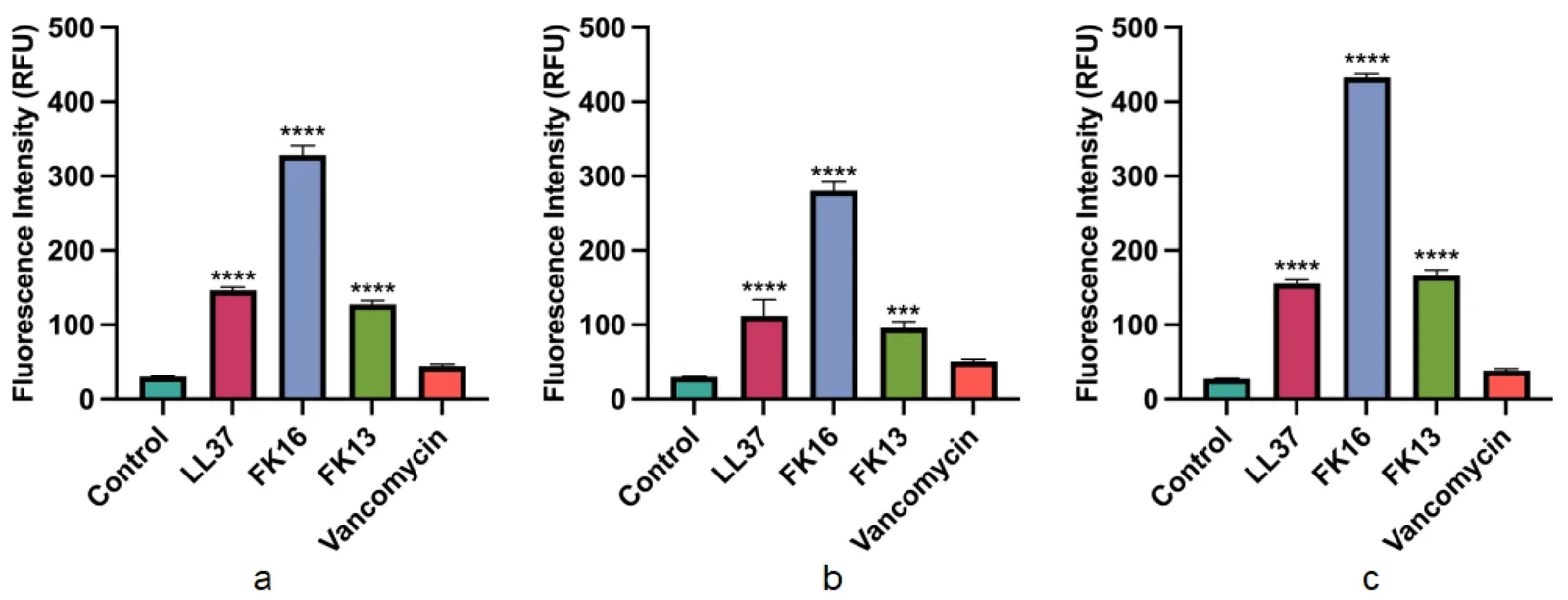
Cytoplasmic membrane permeabilizing properties of antimicrobials to (**a**) *E. coli* B78, (**b**) *E. coli* B37, (**c**) *E. coli* B73. The error bars refer to the standard deviations after three independent experiments. *** stands for *p* ≤ 0.001, **** stands for *p* ≤ 0.0001.

FK16 exhibited the strongest cytoplasmic membrane permeabilizing activity among the three AMPs, consistent with its lower MICs and enhanced antibacterial efficacy (Figure 10). LL37 and FK13 showed similar, moderate permeabilizing effects, which align with their comparable antibacterial performance. Vancomycin, while capable of acting on the cytoplasmic membrane, showed limited activity in this assay, likely due to poor outer membrane penetration.

### 3.7. Cytotoxicity of AMPs Toward Human Dermal Fibroblasts

**Figure 11:**
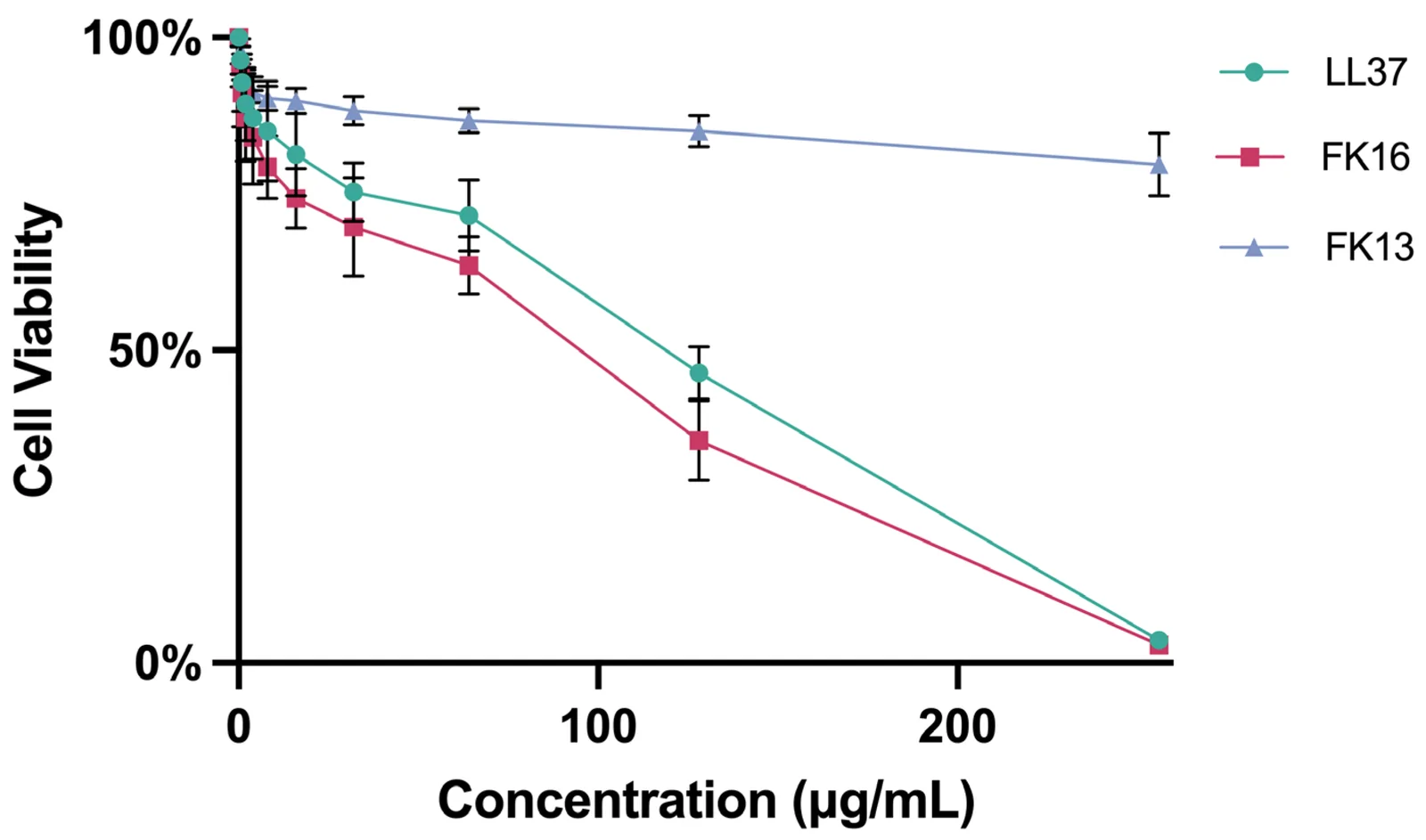
Effect on cell viability for LL37, FK16, and FK13 against HDF cells. The error bars indicate the standard deviations after three independent experiments. The *x*-axis represents increasing peptide concentrations (μg/mL) and the *y*-axis represents cell viability.

LL37 and FK16 exhibited significant cytotoxicity toward human dermal fibroblasts (HDFs) at high concentrations, with nearly complete cell death observed at 256 μg/mL (Figure 11). In contrast, FK13 showed markedly lower cytotoxic effects, suggesting improved biocompatibility and greater clinical potential. The reduced toxicity of FK13 may be due to the absence of hydrophobic residues N30, L31, and V32, which are present in LL37 and FK16 and have been associated with cytotoxicity.

## 4. Discussion

The development of drug resistance poses a major threat to public health worldwide, driving up healthcare costs, hospitalizations, and mortality rates [33]. Considering the challenges in launching new antibiotics to market, novel antimicrobials and therapeutic strategies are urgently needed. One promising clinical approach is the combination of two or more antimicrobials, particularly for treating resistant infections [11,34]. Antimicrobial peptides (AMPs) represent a novel class of therapeutic candidates with potent antibacterial activity [1]. In this study, we investigated the synergistic potential of LL37 and its fragments (FK16 and FK13) in combination with vancomycin, polymyxin B, and colistin against three clinical *E. coli* strains, while also examining the underlying antibacterial mechanisms of these AMPs.

Synergy between antimicrobials with different targets is an attractive strategy [35,36,37]. Vancomycin, which binds to lipid II, is generally ineffective against Gram-negative bacteria due to the outer membrane barrier, but combining vancomycin with outer membrane-permeabilizing agents, such as AMPs, may enhance its uptake and activity [17,21]. In our study, a few synergistic combinations between vancomycin and AMPs were observed, though overall synergy was limited, and the concentrations required were relatively high. These results highlight the need for further investigation into optimizing AMP-vancomycin combinations.

In contrast, AMP combinations with polymyxin B and colistin showed better synergy, especially under cation-adjusted conditions. Polymyxin B and colistin disrupt both outer and cytoplasmic membranes via the self-promoted uptake pathway [38], potentially facilitating AMP access to intracellular targets. LL37, in particular, is known to affect bacterial metabolism and energy production in addition to membrane disruption [39]. Whether FK16 and FK13 share these intracellular targets remains to be determined. An additional benefit of AMP-polymyxin combinations is the potential to reduce polymyxin dosing, thereby minimizing nephrotoxicity and neurotoxicity [40,41]. Given the reintroduction of polymyxins into clinical use due to rising resistance, the ability to lower toxic doses through synergy can present an important clinical advantage.

LPS is a key structural component of the Gram-negative outer membrane and contributes to its negative surface charge. We showed that LL37 and its fragments neutralize the bacterial surface charge via electrostatic interactions with LPS, as reflected in zeta potential shifts. The reduced AMP activity in the presence of exogenous LPS supports the hypothesis that free LPS can bind and sequester AMPs, limiting their availability for membrane targeting. LPS binding is also important for the immunomodulatory roles of AMPs, such as endotoxin neutralization and sepsis prevention [31,32,42,43,44]. LL37 has demonstrated both antimicrobial and anti-sepsis potential, although whether FK16 and FK13 retain these properties requires further study. It has to be noted that only a single, relatively high concentration of exogenous LPS (100 μg/mL) was tested. While this concentration served as a stringent condition to evaluate the maximal inhibitory effect of LPS on AMP activity, future studies incorporating a range of lower, more physiologically relevant LPS concentrations are needed to determine the threshold at which AMP activity becomes significantly impaired.

Charge neutralization strongly correlates with the net positive charge of the antimicrobial. LL37, with the highest net charge (+6), required the lowest concentration to neutralize bacterial zeta potentials, followed by polymyxin B (+5) and FK peptides (+4). Ampicillin, with a net charge of 0, showed no such effect. These results align with previous studies showing that cationic agents increase membrane permeability through charge neutralization [9,21,22,30,45,46]. For example, CTAB increases outer membrane permeability by disrupting surface charge [45]. This interaction may interfere with the cell’s ability to maintain a stable zeta potential, altering membrane integrity and leading to cell death [45,47,48]. In our study, the extent of charge neutralization generally corresponded with outer membrane permeabilization in the absence of divalent cations, reinforcing this relationship.

Pretreating *E. coli* with AMPs sensitized the bacteria to vancomycin, further supporting the role of the outer membrane as a permeability barrier [21,49]. Although vancomycin-AMP synergy was limited, all three AMPs were able to reduce vancomycin resistance across strains. These results, along with prior findings in *P. aeruginosa* [21], support the strategy of using outer membrane-disrupting agents to expand the utility of vancomycin against Gram-negative pathogens.

The role of Mg^2+^ and Ca^2+^ in stabilizing the outer membrane was also confirmed. These cations bind LPS to form salt bridges, reducing membrane permeability and increasing MICs for cationic antimicrobials [50]. Our data show that AMP efficacy declined in the presence of divalent cations, consistent with reduced permeability and electrostatic competition. Higher AMP concentrations may be needed to displace these cations and achieve effective membrane coverage [28,51,52,53,54]. The observation that polymyxin B was least affected by the presence of Mg^2+^ and Ca^2+^ among the tested antimicrobials may be explained by their mechanisms of interaction with LPS. The linear cationic peptides (LL-37, FK16, and FK13) rely primarily on electrostatic interactions with the negatively charged phosphate groups of LPS [55]; the presence of divalent cations competes for these binding sites, thereby reducing peptide binding and activity. Colistin, while possessing a cyclic lipopeptide structure that provides some hydrophobic anchoring, still depends largely on electrostatic interactions and thus remains sensitive to divalent cations. In contrast, polymyxin B possesses a slightly longer fatty acyl chain (e.g., 6-methyloctanoyl in polymyxin B vs. 6-methylheptanoyl in colistin) [56], which may enhance its hydrophobic insertion into the lipid A region [57]. This stronger hydrophobic interaction likely compensates for the charge-shielding effect of Mg^2+^ and Ca^2+^, rendering polymyxin B activity less sensitive to the presence of divalent cations [58].

Outer membrane permeabilization alone was not sufficient for antibacterial activity. LL37 had the strongest permeabilizing effect but was not the most effective antimicrobial. Instead, cytoplasmic membrane disruption appears to be more predictive of bactericidal activity [27,59,60,61,62,63,64]. FK16, which showed the highest cytoplasmic membrane permeabilization, also had the strongest antibacterial effects. LL37 and FK13 displayed similar cytoplasmic membrane activity, aligning with their comparable MICs. As cationic peptides, LL37 and its fragments may disrupt the cytoplasmic membrane by targeting LPS or other negatively charged components [65]. Future work is needed to identify their specific binding sites.

This study is a mechanistic proof-of-concept investigation. Combination cytotoxicity was not tested, as it falls outside the scope of this work and would require a separate translational study. While we acknowledge that the therapeutic window of these combinations remains to be determined, future studies should systematically assess the combination cytotoxicity using mammalian cell lines.

## 5. Conclusions

This study highlights the potential of polymyxin B and colistin in combination with AMPs—particularly LL37 and its fragments—as a promising strategy to combat antibiotic-resistant *E. coli*. These combinations exhibited strong synergistic effects, especially under physiologically relevant conditions. We confirmed that outer membrane permeabilization is closely linked to antimicrobial charge, and that divalent cations such as Mg^2+^ and Ca^2+^ stabilize the membrane by interfering with this process. Additionally, we demonstrated that cytoplasmic membrane permeabilization is a key determinant of AMP efficacy. Together, these findings support the continued exploration of AMP-antibiotic combinations and guide future efforts to optimize AMP design for improved potency, selectivity, and clinical applicability.

## Figures and Tables

**Table 1:** Net charges of different antimicrobials at neutral pH.

Antimicrobials	Net Charge
LL37	+6
FK16	+4
FK13	+4
Polymyxin B	+5
Vancomycin	+0.83 [26]
Ampicillin	0

**Table 2:** MICs of AMPs and different antibiotics against *E. coli* strains in MHB medium.

Antimicrobials	MIC (μg/mL)		
	*E. coli* B78	*E. coli* B37	*E. coli* B73
LL37	16	32	16
FK16	8	4	2
FK13	32	16	16
Vancomycin	64	64	64
Polymyxin B	0.125	0.0625	0.0625
Colistin	0.0625	0.0625	0.0625

**Table 3:** MICs of AMPs and different antibiotics against *E. coli* strains in CMHB medium.

Antimicrobials	MIC (μg/mL)		
	*E. coli* B78	*E. coli* B37	*E. coli* B73
LL37	64	>256	64
FK16	32	64	16
FK13	128	64	64
Vancomycin	128	128	128
Polymyxin B	0.125	0.125	0.125
Colistin	0.125	0.125	0.0625

**Table 4:** MICs of AMPs and different antibiotics against *E. coli* strains in MHB medium with addition of 100 μg/mL LPS.

Antimicrobials	MIC (μg/mL)		
	*E. coli* B78	*E. coli* B37	*E. coli* B73
LL37	>256	>256	>256
FK16	64	16	16
FK13	128	128	128

**Table 5:** Combined activity of AMPs with antibiotics against *E. coli* strains in MHB medium.

Strain	∑ FICI			
	Antimicrobials	Vancomycin	Polymyxin B	Colistin
*E. coli* B78	LL37	>0.5	**0.5**	**0.5**
	FK16	>0.5	**0.5**	>0.5
	FK13	**0.5**	**0.5**	>0.5
*E. coli* B37	LL37	**0.5**	**0.5**	**0.5**
	FK16	>0.5	>0.5	>0.5
	FK13	>0.5	>0.5	>0.5
*E. coli* B73	LL37	>0.5	**0.5**	**0.5**
	FK16	>0.5	>0.5	**0.5**
	FK13	**0.3125**	**0.5**	**0.5**

**Table 6:** Combined activity of AMPs with antibiotics against *E. coli* strains in CMHB medium.

Strain	∑ FICI			
	Antimicrobials	Vancomycin	Polymyxin B	Colistin
*E. coli* B78	LL37	>0.5	**0.375**	**0.375**
	FK16	>0.5	**0.5**	**0.5**
	FK13	>0.5	**0.375**	**0.375**
*E. coli* B37	LL37	**<0.375**	**<0.5**	**<0.5**
	FK16	**0.5**	**0.375**	**0.5**
	FK13	>0.5	**0.5**	**0.5**
*E. coli* B73	LL37	>0.5	**0.5**	>0.5
	FK16	>0.5	**0.375**	>0.5
	FK13	>0.5	**0.5**	>0.5

**Table 7:** Sensitization of *E. coli* strains to vancomycin after treatment with LL37, FK16, and FK13.

Strain	AMPs	Fold of Sensitization (MIC_Control_/MIC_Treated_)
*E. coli* B78	LL37	24
	FK16	4
	FK13	4.4
*E. coli* B37	LL37	32
	FK16	1.7
	FK13	3.8
*E. coli* B73	LL37	2.4
	FK16	1.7
	FK13	2.2

## Data Availability

The original contributions presented in this study are included in the article. Further inquiries can be directed to the corresponding authors.

## Supplementary Materials

The following supporting information can be downloaded at: https://www.mdpi.com/article/10.3390/biom16091324/s1. Table S1. MICs of antimicrobials alone and in combination against *E. coli* strains with corresponding FICI values in MHB medium. All MIC values are expressed in μg/mL; Table S2. MICs of antimicrobials alone and in combination against *E. coli* strains with corresponding FICI values in CMHB medium. All MIC values are expressed in μg/mL.

## Abbreviations

The following abbreviations are used in this manuscript:

AMPs	Antimicrobial peptides
*E. coli*	*Escherichia coli*
AMR	Antimicrobial resistance
WHO	World Health Organization
VIM-GES-CRPA	Verona Integron-encoded Metallo-β-lactamase—Guiana Extended Spectrum—Carbapenem-Resistant *Pseudomonas aeruginosa*
LPS	lipopolysaccharide
MHB	Mueller–Hinton broth
MHA	Mueller–Hinton agar
IMDM	Iscove’s Modified Dulbecco’s Medium
FBS	Fetal Bovine Serum
DPBS	Dulbecco’s phosphate-buffered saline
HEPES	4-(2-hydroxyethyl) piperazine-1-ethanesulfonic acid
NPN	1-N-phenylnaphthylamine
PI	Propidium iodide
MIC	Minimum inhibitory concentrations
CLSI	Clinical and Laboratory Standards Institute
FICI	Fractional inhibitory concentration index
