# Supplementary material for: Membrane Disruption and Charge Neutralization Drive Synergy Between LL37-Derived Peptides and Polymyxins Against E. coli
Source: Biomolecules. 2026 Sep 11;16(9):1324. doi: 10.3390/biom16091324 (PMC13604721; doi:10.3390/biom16091324)
Supplement: Supplementary file 1 [file biomolecules-16-01324-s001.zip › biomolecules-4481089-supplementary.pdf]

Supplementary Material

Note: The values under "MIC of A/B used alone" are the MICs of antimicrobial A and antimicrobial B when used alone, respectively. Correspondingly, the values under "MIC of A/B when used in combination" are the MICs of these two agents when used in combination, respectively. Both values are essential for the standard FICI calculation, which requires the ratio for each drug individually: FICI = (MIC of A in combination / MIC of A alone) + (MIC of B in combination / MIC of B alone).

Table S1. MICs of antimicrobials alone and in combination against *E. coli* strains with corresponding FICI values in MHB medium. All MIC values are expressed in μg/mL.

Strain	Combination (A and B)	MIC of A when used alone	MIC of B when used alone	MIC of A when used in combination	MIC of B when used in combination	FICI
E. coli B78	LL-37 and Vancomycin	16	64	4	32	> 0.5
	FK16 and Vancomycin	8	64	2	32	> 0.5
	FK13 and Vancomycin	32	64	8	16	**0.5**
	LL-37 and Polymyxin B	16	0.125	4	0.03125	**0.5**
	FK16 and Polymyxin B	8	0.125	2	0.03125	**0.5**
	FK13 and Polymyxin B	32	0.125	8	0.03125	**0.5**
	LL-37 and Colistin	16	0.0625	4	0.015625	**0.5**
	FK16 and Colistin	8	0.0625	4	0.03125	> 0.5
	FK13 and Colistin	32	0.0625	16	0.03125	> 0.5
E. coli B37	LL-37 and Vancomycin	32	64	8	16	**0.5**
	FK16 and Vancomycin	4	64	2	32	> 0.5
	FK13 and Vancomycin	16	64	8	16	> 0.5
	LL-37 and Polymyxin B	32	0.0625	8	0.015625	**0.5**
	FK16 and Polymyxin B	4	0.0625	1	0.03125	> 0.5
	FK13 and Polymyxin B	16	0.0625	8	0.03125	> 0.5

	LL-37 and Colistin	32	0.0625	8	0.015625	**0.5**
	FK16 and Colistin	4	0.0625	2	0.03125	> 0.5
	FK13 and Colistin	16	0.0625	8	0.03125	> 0.5
E. coli B73	LL-37 and Vancomycin	16	64	8	16	> 0.5
	FK16 and Vancomycin	2	64	1	16	> 0.5
	FK13 and Vancomycin	16	64	4	4	**0.3125**
	LL-37 and Polymyxin B	16	0.0625	4	0.015625	**0.5**
	FK16 and Polymyxin B	2	0.0625	1	0.03125	> 0.5
	FK13 and Polymyxin B	16	0.0625	4	0.015625	**0.5**
	LL-37 and Colistin	16	0.0625	4	0.015625	**0.5**
	FK16 and Colistin	2	0.0625	0.5	0.015625	**0.5**
	FK13 and Colistin	16	0.0625	4	0.015625	**0.5**

Table S2. MICs of antimicrobials alone and in combination against *E. coli* strains with corresponding FICI values in CMHB medium. All MIC values are expressed in µg/mL.

Strain	Combination (A and B)	MIC of A when used alone	MIC of B when used alone	MIC of A when used in combination	MIC of B when used in combination	FICI
E. coli B78	LL-37 and Vancomycin	64	128	32	32	> 0.5
	FK16 and Vancomycin	32	28	8	64	> 0.5
	FK13 and Vancomycin	128	128	32	64	> 0.5
	LL-37 and Polymyxin B	64	0.125	8	0.03125	**0.375**
	FK16 and Polymyxin B	32	0.125	8	0.03125	**0.5**
	FK13 and Polymyxin B	128	0.125	16	0.03125	**0.375**
	LL-37 and	64	0.125	8	0.03125	**0.375**

	Colistin					
	FK16 and Colistin	32	0.125	8	0.03125	**0.5**
	FK13 and Colistin	128	0.125	16	0.03125	**0.375**
E. coli B37	LL-37 and Vancomycin	> 256	128	64	16	**< 0.375**
	FK16 and Vancomycin	64	128	16	32	**0.5**
	FK13 and Vancomycin	64	128	32	32	> 0.5
	LL-37 and Polymyxin B	> 256	0.125	64	0.03125	**< 0.5**
	FK16 and Polymyxin B	64	0.125	16	0.015626	**0.375**
	FK13 and Polymyxin B	64	0.125	16	0.03125	**0.5**
	LL-37 and Colistin	> 256	0.125	64	0.03125	**< 0.5**
	FK16 and Colistin	64	0.125	16	0.03125	**0.5**
	FK13 and Colistin	64	0.125	16	0.03125	**0.5**
E. coli B73	LL-37 and Vancomycin	64	128	32	32	> 0.5
	FK16 and Vancomycin	16	128	8	32	> 0.5
	FK13 and Vancomycin	64	128	32	32	> 0.5
	LL-37 and Polymyxin B	64	0.125	16	0.03125	**0.5**
	FK16 and Polymyxin B	16	0.125	4	0.015625	**0.375**
	FK13 and Polymyxin B	64	0.125	16	0.03125	**0.5**
	LL-37 and Colistin	64	0.0625	32	0.015625	> 0.5
	FK16 and Colistin	16	0.0625	8	0.015625	> 0.5
	FK13 and Colistin	64	0.0625	32	0.015625	> 0.5
